# Manufacturing readiness assessment for evaluation of the microneedle array patch industry: an exploration of barriers to full-scale manufacturing

**DOI:** 10.1007/s13346-021-01076-4

**Published:** 2021-10-15

**Authors:** Ben Creelman, Collrane Frivold, Sierra Jessup, Gene Saxon, Courtney Jarrahian

**Affiliations:** PATH (Medical Devices and Health Technologies Program), Seattle, WA USA

**Keywords:** Microneedle patch, Scale-up, Manufacturing barriers, Quality control, Critical quality attributes, Production

## Abstract

Microneedle array patch (MAP) technology is a promising new delivery technology for vaccines and pharmaceuticals, yet due to several differing and novel production methods, barriers to full-scale manufacturing exist. PATH conducted a manufacturing readiness assessment and follow-up interviews to identify both the current manufacturing readiness of the industry as well as how readiness varies by developer type and MAP type. Follow-up interviews identified barriers the industry faces in reaching full manufacturing readiness, including the perceived regulatory and investment risk of manufacturing MAPs at scale due to quality requirements and control methods, uncertain sterility requirements, lack of standard production methods (especially around dissolvable MAP drying methods), and the lack of available contract manufacturing organizations with MAP manufacturing capabilities. A Regulatory Working Group has been established to identify and address critical quality issues specific to MAP manufacturing with the aim of providing developers insight into what will be expected for MAP product approvals. Standardizing MAP production equipment and automatic, visual quality control could reduce the overall investment risk to developers and contract manufacturing organizations in pursuing pilot-scale manufacturing capabilities and ultimately lower barriers to the scale-up of full medical MAP product lines.

**Graphical abstract:**

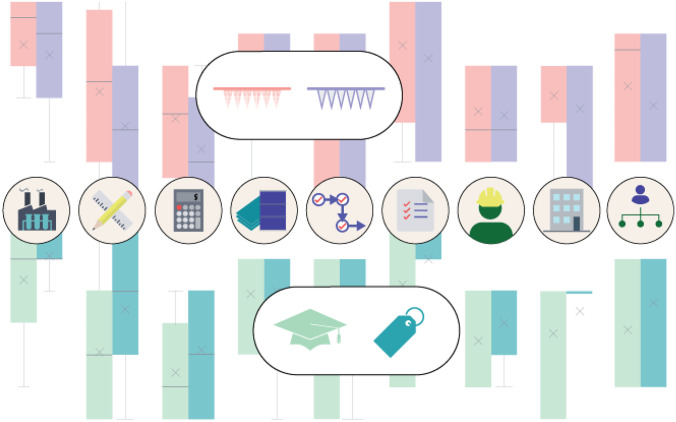

## Introduction

Microneedle array patches (MAPs), also referred to as microneedle patches, offer an alternative to injectable methods of administering drugs and biologics [1]. A MAP contains tens to thousands of projections less than 1 mm long that are either coated with or composed of a dry formulation and designed to puncture the stratum corneum. There are several MAP types in development, including solid-coated, dissolving, and hydrogel that aim to administer a vaccine or drug to the dermis or epidermis. Organizations advancing pharmaceutical MAP technology include academic groups and biotechnology companies of varying sizes. The pharmaceutical industry has also expressed interest in MAPs. For instance, Merck & Co., Inc., has announced a partnership with Vaxxas Inc. to develop a vaccine MAP [2], and Serum Institute of India has contributed material for MAP clinical studies for both measles-rubella and hepatitis B vaccines.

At the time of this evaluation, production-scale manufacturing facilities exist only for cosmetic MAP products [3]. MAPs for the delivery of drugs and vaccines are in either preclinical or clinical development, and commercial-scale manufacturing of pharmaceutical MAPs is still, on the whole, in its infancy. One drug-delivery MAP for zolmitriptan (for migraine headache), developed by Zosano Pharma, has completed a phase 3 study [4]. However, the US Food and Drug Administration requested additional data in response to the company’s New Drug Application for its Qtrypta™ MAP for migraine treatment due to inconsistent drug exposure levels in study recipients who used MAPs from different lots of the product, a setback for achieving regulatory approval of the first pharmaceutical MAP product in the USA [5]. A phase 3 study for an abaloparatide MAP to treat osteoporosis from Radius Pharma is currently ongoing [6, 7]. Several phase 1 studies have also been completed for seasonal influenza vaccine MAPs [8–11].

Many pharmaceutical MAP developers are using manual, lab-scale MAP fabrication processes not suitable for mass production. Since MAP manufacturing methods are not standardized and are highly dependent on MAP design, formulation requirements, and anticipated use case, production-scale manufacturing will require development of custom equipment and novel processes. A recent partnership between Harro Höfliger, a leading MAP production equipment development firm, and Vaxxas is aimed at developing the first high-throughput aseptic production line for solid-coated vaccine MAPs [12]. Each line will have a targeted throughput of up to 5 million units per week. Employing a modular manufacturing approach with multiple production lines would facilitate production of tens of millions of units per week. These targets suggest that it could be feasible and economical to produce MAPs at scale. However, stringent regulatory and manufacturing requirements for these types of medical products without well-established precedents will be a hurdle to the scale-up of vaccine and drug MAPs.

MAPs have the potential to foster substantial gains in access and adherence to vaccines and essential medicines and to reduce burdens on strained health systems, particularly in low- and middle-income countries where there is a shortage of health workers trained to give injections. This technology has been identified by the Vaccine Innovation Prioritisation Strategy Alliance—a collaboration between the Gavi Secretariat, World Health Organization, Bill & Melinda Gates Foundation, United Nations Children’s Fund, and PATH—as a priority innovation for advancement of development, policy, and access [13]. PATH’s MAP Center of Excellence was established as a key resource to provide leadership and guidance to the MAP technology field in advancing product development, understanding public health needs, reviewing manufacturing technologies, defining regulatory pathways, and demonstrating commercial viability [14]. A key focus area of the MAP Center of Excellence has been on manufacturing, and to this end, PATH co-hosted a 3-day MAP Manufacturing Workshop in collaboration with Harro Höfliger at its facility in Germany in January 2020. The Center aims to identify gaps in manufacturing readiness and investment risks associated with MAP manufacturing to inform both strategy for maturation of manufacturing technologies and future investment opportunities. Although the needs of the MAP product development process are fairly well-understood, production readiness for MAP manufacturing has not been formally evaluated. Therefore, PATH conducted a manufacturing readiness assessment to provide industry stakeholders with information on the status of MAP manufacturing, general barriers to manufacturing scale-up, and potential approaches for mitigating these issues to help inform future investment in and development of the industry. This paper outlines the results of that assessment.

## Methods

This assessment consisted of both an online survey as well as individual follow-up interviews for added depth of information to quantify manufacturing readiness. To determine individual and aggregate levels of manufacturing readiness in the pharmaceutical MAP industry, an online survey was distributed via email in December 2019 to academic and commercial developers. Potential participants with direct involvement in MAP manufacturing were identified from literature reviews, internet searches, and PATH’s database on technology developers related to MAP innovations. A manufacturing readiness assessment toolkit or “deskbook” [15] developed by the United States Department of Defense was used to develop the survey questions, which were modified as appropriate to ensure relevance to developers of MAP technology.

The survey was used to identify the manufacturing readiness level (MRL) of each developer across various manufacturing categories. The ten MRLs are defined as follows:

- Level 1: Basic manufacturing implications identified
- Level 2: Manufacturing concepts identified
- Level 3: Manufacturing proof of concept developed
- Level 4: Capability to produce the technology in a laboratory environment
- Level 5: Capability to produce prototype components in a production relevant environment
- Level 6: Capability to produce a prototype system or subsystem in a production relevant environment
- Level 7: Capability to produce systems, subsystems, or components in a production representative environment
- Level 8: Pilot line capability demonstrated; ready to begin low-rate initial production
- Level 9: Low-rate production demonstrated; capability in place to begin full-rate production
- Level 10: Full-rate production demonstrated and lean production practices in place [15]

In line with the Department of Defense toolkit, each question in this survey corresponded to a distinct MRL in one of the following nine manufacturing categories:

- *Technology and industrial base*
- *Design*
- *Cost and funding*
- *Materials*
- *Process capability and control*
- *Quality management*
- *Manufacturing personnel*
- *Facilities*
- *Manufacturing management*

Note: “Design” also includes all development tasks related to formulation development, array performance, and delivery device performance.

For this survey and analysis, each category was represented by a series of questions representing progressively higher MRLs. To ensure the survey was manageable and to improve completion rates, a total of 45 questions related to a subset of MRLs most relevant to MAP development status were selected. For example, no questions related to an MRL of 10 in any category were asked because it is common knowledge that no developer is at that stage with MAP technology. During the analysis phase, each developer was assigned an MRL for each category equivalent to the highest level question answered in the affirmative in that category. Afterwards, the results from all respondents were aggregated to ensure anonymity.

As a follow-up to the online survey, the study team conducted in-depth qualitative interviews in February and March of 2020 with a subset of respondents chosen based on the following categories: most and least advanced (based on MRA results), singularly focused on MAP products, and diversely focused (have products other than MAPs). To help identify gaps in manufacturing readiness, interviews were focused on technical questions related to the developer’s specific MAP technology, including questions designed to better understand the manufacturing categories where developers scored the lowest MRL. Other interview topics were related to future manufacturing scale-up plans, resources, and timelines for MAP production. These activities were determined to be not human subjects research as defined by the US Department of Health & Human Services regulations in 45 CFR 46.102 by the Research Determination Committee of PATH’s Office of Research Affairs.

## Results

The survey was distributed to 78 MAP developer organizations, and 27 completed the survey (34.6% response rate), representing 10 countries. The majority of respondents were commercial organizations (75%), with the remainder from academic organizations. Respondents included developers working on solid-coated, dissolving, and hydrogel MAP subtypes. Of the respondents, 58% and 37% indicated that their most advanced MAP product was a dissolving MAP or solid-coated MAP, respectively, with one organization electing not to respond. Of the commercial respondents, 7 claimed solid-coated MAPs as their most advanced MAP and 11 claimed dissolving; of the academic respondents, 2 claimed solid-coated MAPs and 6 claimed dissolving. Although some developers are advancing hydrogel MAPs, none of the respondents indicated that it was their most advanced MAP product.

The survey illustrated that manufacturing readiness varied among MAP developers and assessment categories, with MRLs ranging from manufacturing concept/proof-of-concept stages (MRLs 2 and 3) to demonstrated pilot line capability (MRL 8).

An aggregate summary of average MRL ratings and MAP-specific interpretations for each manufacturing category based on all developer responses is shown in Table 1. The *technology and industrial base* and *quality management* categories tied for the highest average score (6.6), whereas the *cost and funding* category had the lowest average score (4.0).

**Table 1:** Average manufacturing readiness level for nine manufacturing categories: scores and interpretation

**Manufacturing category**	**Mean manufacturing readiness level** Scale: 1 to 10 *N* = 27 developers	**Interpretation**^**a**^
*Technology and industrial base*	6.6	Industrial capabilities in place to support manufacturing of prototype devices in a production relevant environment
*Design*	5.0	Preliminary design work in progress and able to support evaluation of manufacturability
*Cost and funding*	4.0	Processes, materials, and designs can provide reasonable estimates, including capital expenditures
*Materials*	5.9	Preliminary material specifications are in place and supply chain capacity is identified
*Process capability and control*	4.9	Pilot line processes are identified at the component level
*Quality management*	6.6	Quality plan and system is in place. Inspection processes and acceptance criteria are identified
*Manufacturing personnel*	4.6	Manufacturing skill sets are identified, and production workforce requirements evaluated
*Facilities*	4.9	Manufacturing facilities are being evaluated and plans developed for prototype production
*Manufacturing management*	5.1	Manufacturing strategy is refined based on preferred design concept. Make/buy evaluations initiated and include production considerations for pilot line

^a^Adapted from [15]

**Fig. 1:**
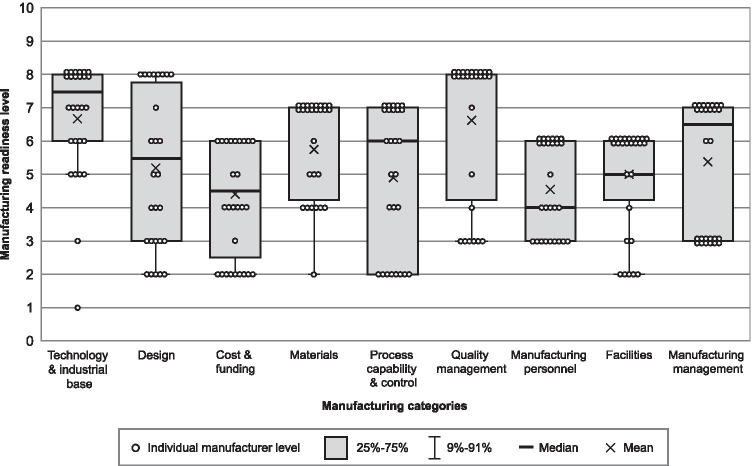
Summary of manufacturing readiness assessment results from all respondents (*N* = 27)

A summary of all assessment responses (academic and commercial developers combined) is shown in box plot format in Fig. 1. This figure demonstrates the distribution of manufacturer readiness levels for each category. Among all developers, the *design* category and the *process capability and control* category represent the largest quartile spread in manufacturing readiness, whereas on average, *technology and industrial base* and *quality management* were reported to be the furthest advanced categories. *Cost and funding*, notably, had the lowest reported average of all categories.

**Fig. 2:**
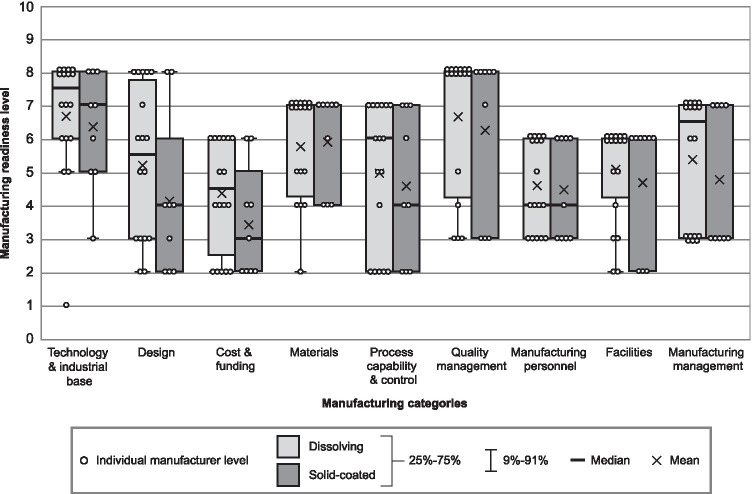
Manufacturing readiness assessment results for solid-coated vs. dissolving microneedle array patch (*N* = 27)

Figure 2 illustrates the difference in responses for the two MAP types reported as most advanced in this survey: solid-coated and dissolving. For all manufacturing categories except for *materials*, developers with solid-coated MAPs as their primary focus self-reported being less advanced on average (mean) than those focusing on dissolving MAPs. For both MAP types, the interquartile ranges of MRLs for the *process capability and control*, *manufacturing personnel*, and *manufacturing management* categories were comparable.

**Fig. 3:**
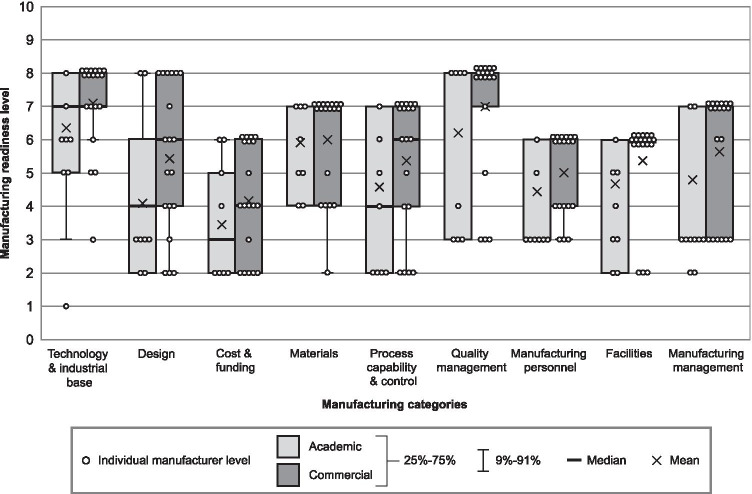
Manufacturing readiness assessment results per microneedle array patch developer type (*N* = 27)

The responses by developer type (academic vs. commercial) are shown in Fig. 3. Commercial developers reported a more advanced mean MRL in every category. On average, academic developers had larger interquartile ranges in MRLs than commercial developers.

Follow-up interviews with developers explored further the results of the survey and identified areas in need of additional development, including quality requirements, quality control methods, sterility requirements, and aseptic manufacturing complexity. The interviews highlighted a lack of contract manufacturing organizations (CMOs) well-positioned to transition MAP technologies to pilot- and commercial-scale production levels. Regarding sterility, manufacturers noted specifically that MAPs with extended drying times face significant manufacturing complexity risks if aseptic production is required. Additionally, manufacturers mentioned that securing bulk active pharmaceutical ingredients to test in MAP prototypes was proving difficult, sometimes due to a lack of confidence in MAPs in the pharmaceutical industry.

### MAP Manufacturing Workshop

The results of the survey informed the preparations for a 3-day “MAP Manufacturing Workshop” PATH hosted in collaboration with Harro Höfliger at its facility in Germany in January 2020. In total, 75 individuals participated representing 37 organizations. The workshop brought together MAP developers and key stakeholders to share learning about scaling MAP manufacturing from lab to production and to identify shared manufacturing challenges and investment risks. The workshop was also an opportunity to disseminate information related to manufacturing scale-up as well as inform future MAP Center of Excellence activities by identifying key manufacturing barriers and potential solutions.

During the workshop, unique challenges of MAP platform production were highlighted, which aligned with the results of this manufacturing readiness survey, including quality and regulatory issues (such as imaging-based, in-line quality control processes, and the implications of aseptic production). Open-source information on solutions to common challenges, such as drying time and automatic visual quality control systems, were suggested as potential areas for future focus, as well as applicator/indicator technologies.

## Discussion

Self-reported manufacturing readiness varied considerably among MAP developers, with MRLs ranging from early manufacturing concepts to advanced pilot line capability. Readiness also varied for different manufacturing categories, with developers generally reporting the highest MRLs in *technology and industrial base*, *quality management*, and *materials* categories, whereas the *cost and funding*, *manufacturing personnel*, and *process capability and control* categories were generally in earlier stages.

It is notable that although the few pharmaceutical MAP products that are closest to licensure are mainly of the solid-coated type, the average readiness for dissolving MAP technology was found to be either equal to, or more advanced than, solid-coated MAP technology in all manufacturing categories. This may have been due to the specific solid-coated MAP developers that responded to the survey.

The manufacturing gaps and barriers to scaled manufacturing discussed in follow-up interviews included quality issues, sterility requirements, and CMO availability. These barriers also align with feedback raised by stakeholders during the MAP Manufacturing Workshop.

### Quality requirements and quality control methods

A major barrier to entry for MAP developers is that this product class is lacking precedent, with minimal guidance documentation available for the design, development, and testing of MAPs; therefore, developers need to defend their own performance requirements for each new product. Prior to and during establishment of manufacturing facilities, Chemistry, Manufacturing, and Controls (CMC) work is needed to establish MAP-specific pharmaceutical formulations and to define and establish test methods for key quality parameters, including ensuring uniformity of product and dose delivery. Through the MAP Center of Excellence, PATH has partnered with Cardiff University in Wales to establish a Regulatory Working Group to facilitate collaboration among MAP developers, regulatory authorities, and public health stakeholders in order to define the MAP delivery system and identify critical quality attributes, develop standardized test methods, and evaluate sterility requirements for the technology class [16].

For high-speed production of MAPs, novel technological innovations are likely to be needed to facilitate nondestructive in-line quality control, such as automated visual inspection of micron-scale MAP projections.

### Sterility requirements and aseptic manufacturing

Since MAP technology falls between transdermal patches, which can be produced in low-bioburden environments, and intradermal injection technologies, which must be produced aseptically if not able to be terminally sterilized, it is unclear what level of sterility assurance will be required by regulators for commercial MAP products. The three possible manufacturing routes for MAP products are aseptic manufacturing, low-bioburden manufacturing followed by terminal sterilization, and low-bioburden-only manufacturing. Since some active pharmaceutical ingredients in vaccines and biologics cannot withstand terminal sterilization, developers must decide whether to expend significant resources pursuing aseptic production or instead develop a lower cost, low-bioburden process and risk being denied approval by regulatory authorities if they are unable to demonstrate an acceptable level of safety risk to the end user.

Safety risks associated with low-bioburden manufacturing center around the potential of a MAP to cause a local infection (adverse event), which could lead to complications such as systemic infection. Laboratory-based studies have shown that because MAPs physically disrupt the skin barrier, microorganisms can penetrate beyond the stratum corneum—but not the epidermis (whereas a 21-gauge hypodermic needle has been shown to allow this), suggesting that a local or systemic infection is unlikely from use of MAPs [17]. Due to the lack of clinical precedent, justifying the acceptability of non-aseptic production would require evaluation of clinical data and testing efforts to support a user safety risk analysis, but the end result may be substantial reductions in manufacturing costs. This topic is also the subject of review by the MAP Center of Excellence’s Regulatory Working Group. However, most developers are taking the conservative approach of using aseptic manufacturing for MAPs that cannot be terminally sterilized.

Two complexities associated with aseptic manufacturing are the sterility requirements for MAP component materials that must be introduced into the production line (e.g., formulations, molds, packaging) and more extensive sterile product monitoring requirements, both of which are anticipated to increase the manufacturing costs [18]. Thus, significant investment in manufacturing would likely be required to scale up MAP manufacturing for late-stage clinical trials and/or production manufacturing [1, 19].

The drying process is also complicated by potential aseptic requirements—particularly for dissolving MAPs. Compared with solid-coated MAPs that can be dried rapidly (due to their comparatively small liquid volume), the larger liquid volume of dissolving MAPs requires longer drying times to form the microprojections that encapsulate the active pharmaceutical ingredient. If the manufacturer uses a continuous production line to achieve high production volumes, the drying step could significantly increase the manufacturing floor space and number of isolators required to maintain an aseptic environment during drying as well as increase the risk of product loss as a result of line shutdowns. During the in-depth qualitative interviews, survey respondents focusing on dissolving MAPs identified drying as a significant design challenge. Therefore, continued research in this area should be prioritized.

### Contract manufacturing organization availability

For traditional injectable pharmaceutical packaging technologies, such as vials and prefilled syringes, there are hundreds of CMOs available to implement both pilot- and production-scale filling runs using standardized filling equipment designed for these delivery devices. This significantly decreases the capital requirements for developers when engaging in early development and testing efforts. However, during the developer interviews, respondents commented that there were very few, if any, CMOs capable of supporting clinical trials with production of MAPs at a level of quality consistent with good manufacturing practice guidelines. Several barriers impede CMOs from pursuing MAP manufacturing capabilities. First, there is a risk that CMOs may invest resources in a production line for a MAP type (e.g., solid-coated vs. dissolving) that is not prioritized by developers. In addition, since turnkey production equipment is not available for most MAP technologies, it is difficult for CMOs to design manufacturing lines without established development partners. Finally, installing a MAP manufacturing line requires significant time, space, and finances that could be used on more reliable product lines (such as vial filling or blow-fill-seal manufacturing). These requirements would be even higher if aseptic manufacturing is required.

These issues facing CMOs are the same as those facing individual MAP developers; thus, if CMOs are incentivized to develop MAP production capacity, it could defray high initial costs for developers and help accelerate progress.

### Limitations of the survey

The data presented here reflect the manufacturing status of the MAP developers who completed the online survey, which is a portion of known MAP developers. The data are self-reported; as such, the MRLs generated by this analysis are based on the developers’ perspectives and may not reflect their actual status. During the follow-up interviews, efforts were made to validate the online survey responses.

## Conclusion

The PATH MAP Center of Excellence manufacturing readiness assessment survey revealed both the manufacturing readiness of the pharmaceutical MAP developer industry as a whole as well as how it varied between developer types and the prioritized MAP types. Since these survey responses were self-reported, future efforts to independently validate manufacturer readiness may provide additional insight into the state of the industry. Follow-up interviews highlighted key barriers to full production-scale manufacturing that developers face, namely the perceived regulatory and investment risk of manufacturing MAPs at scale due to quality control requirements and methods, uncertain sterility requirements, lack of established large-scale production methods (especially around dissolvable MAP drying), and the lack of availability of CMOs with MAP capabilities. The MAP Regulatory Working Group is working to identify and address key issues specific to developing MAP manufacturing capabilities with the aim of providing developers’ insight into what will be expected for MAP product approvals. Technological advancements in MAP production equipment and automatic visual quality control could benefit many developers within this divergent technology class by enabling more CMOs to support pilot-scale manufacturing, ultimately lowering the barriers to full scale-up of medical MAP production lines.

## Data Availability

Raw data will not be made available due to its confidential nature.
